# Supplementary material for: Pediatric Advance Care Planning: A Scoping Review
Source: Children (Basel). 2023 Jul 7;10(7):1179. doi: 10.3390/children10071179 (PMC10378635; doi:10.3390/children10071179)
Supplement: Supplementary file 1 [file children-10-01179-s001.zip › children-2469149-supplementary.pdf]

Table S1: Summary information for all included studies.

Author, Publication Year, Study origin	Purpose	Study Design	Sample	Findings
Abbott, D. et al., (2017), UK [15]	Aimed to focus on the views and preferences of men with Duchenne Muscular Dystrophy and how they could best be supported to approach end of life planning	Qualitative	15 male participants with DMD, age 20-45	End of life conversations were acknowledged as limited and overly medicalized, none could recount having conversations about this yet. Progression of disease was discussed, but not what end of life would look like. None of the participants had current advance care plans in place. Counselling was accessed for emotional support, conversations with loved ones was limited. Participants stated they would prefer for healthcare providers to bring up conversation topics, be relational in nature, a skilled clinician and to have conversations early and individualized to the needs of the person
Barrison, et al., 2021, USA [16]	To assess the feasibility and efficacy of a novel, peer-led, targeted approach to ACP health engagement through workshops with undergraduate college students	Repeated measures, mixed-methods, survey	74 undergraduate students	Workshop attendance promoted changes in participant confidence and knowledge related to ACP. Increase in participant confidence to discuss ACP with loved ones and increase in knowledge of ACP terms and usefulness. 2 weeks post workshop 11% had competed an advance directive, 33% spoke to loved ones, 50% discussed material with friends
Basu, et al., 2021, USA [14]	To explore multidisciplinary clinician perceptions about perceived barriers and strategies to improve ACP provision	Qualitative – focus groups	34 multidisciplinary participants from a tertiary level children's hospital and cancer institute	Perceived clinician barriers identified – avoidance, prognostic uncertainty, knowledge of the patient/family, multidisciplinary care team dynamics, timing, culture, fear of causing harm and logistics. Perceived patient and parent barriers – avoidance, preferences for receiving information, prognostic awareness, patient involvement, family dynamics. Recommended strategies—asking parental preference for information needs, early initiation of ACP, share prognosis, discuss and plan how to include the child, understand family dynamics, involve palliative care, social work and child life specialists

Author, Publication Year, Study origin	Purpose	Study Design	Sample	Findings
Beecham, et al., 2016, UK [17]	To investigate how parents of children and young people with life limiting conditions (LLC) approach and experience ACP	Qualitative	Parents of 18 children (14 mothers, 4 fathers); 9 children receiving palliative care, 9 who have died and had received palliative care	Most parents had discussions and made decisions around place of care, place of death, limitation of treatment. Decision were usually made late in the illness and parents expressed a preference to want to keep options open. Parents expressed different levels of involvement in a range of decisions and many wished to be involved in decision making but didn't always feel able to. Several parents shared experiences of not being involved at all in some decisions by HCP. Written plans were emphasized by families as important documents to be shared amongst organizations involved
Brown, A., 2019, USA [18]	Presents perspectives on the goals of healing and non-healing rituals, and how reconceived decision making at the end of life in pediatric as a form of healing ritual	Qualitative	5 pediatric emergency physicians, 3 subspecialty pediatricians, 2 paramedics, 5 parents of CMC, 4 health IT specialists	The process of decision-making includes some elements similar to healing rituals in traditional medicine, especially if the family is considered as a potential recipient of healing effects. The use of a framework to consider decision making as part of the healing ritual can attenuate moral distress around end of life decision making and facilitate improved communication and collaboration between parents and clinicians
Conway Copper et al., 2018, USA [19]	To identify factors related to optimal care for children with medical complexity to inform development of emergency information forms	Qualitative	26 healthcare providers (12 pediatric physicians, 2 paramedics, 5 parents, 4 IT and 3 privacy specialists)	Current barriers to care include documentation, poor caregiver understanding, poor provider understanding. Recommended content includes demographic/contact info, medical history, medications, allergies, advanced directives, information about pt. disease, action plan for anticipated emergencies.

Author, Publication Year, Study origin	Purpose	Study Design	Sample	Findings
Coughlin, 2018, Canada [20]	To provide guidance for medical decision making in pediatrics: infancy to adolescence	Position statement	n/a	Discusses ethical principles of medical decision making for pediatric HCPs. Discusses topics such as capacity, assent and dissent, laws regarding age of consent and advance directives for each province, substitute decision makers, best interests, end-of-life decision making, family-centered and shared decision making, withholding/withdrawing life-sustaining interventions, resolving conflicts. Recommendations include child/adolescent participation, HCP should practice in accordance with laws, patient/parents are informed, assent or dissent should be respected, partnered collaborative approach, for conflict seek and access resources for conflict resolution
Curtin et al., 2017, USA [21]	To evaluate the efficacy of Family Centered disease specific advance care planning for teens with cancer (FACES-TC).	Prospective, longitudinal, two arm, randomized controlled single blinded clinical trial	5 pediatric emergency physicians, 3 subspecialty pediatricians, 2 paramedics, 5 parents of CMC, 4 health IT specialists	Methodology of a randomized control trial - results not included in the paper. Described a methodology by which to engage teens with cancer and their family decision maker in advanced care planning process (both the discussion and documentation)
Dallas, et al., 2016, USA [22]	To study the feasibility and acceptability of a pACP intervention in an adequately powered, multisite, randomized clinical trial	Randomized controlled trial	105 adolescent/family dyads participated, adolescent age 14-21 living with HIV	The intervention (Family Centered Advanced Care Planning –FACE) resulted in very high attendance rates, was reported as useful/helpful. Some participants reported feelings of sadness, no associated adverse effects

Author, Publication Year, Study origin	Purpose	Study Design	Sample	Findings
De-Arruda-Colli et al., 2018, Australia [23]	To make a cross cultural adaptation of the advance care planning guide, Voicing my CHOICES, in Australia and Brazil	Mixed methods adaptation	In Brazil, 9 healthcare providers and 15 young adults (19-35 years old), in Australia 27 healthcare providers and 6 AYAs (15-25 years old)	There are important cultural considerations to take into account when translating the tool into another language and applying to a different cultural context. Youth are generally willing and interested to engage in ACP discussions
Decourcey, D., et al., 2021, USA [24]	To develop a generalizable advance care planning intervention for children, adolescents and young adults with serious illness using a multistage, stakeholder driven approach	Mixed methods	9 parents, 7 adolescents, 33 HCPs (18 physicians, 4 nurse practitioners, 6 nurses, 3 SW, 1 RT, 1 psychologist, 1 child life)	The serious illness conversation guide was able to be adapted for pediatrics based on stakeholder input. Identified barriers to PediSCIP implementation included need for HCP training, competing demands, uncertainty regarding timing and documentation of ACP discussions. Patients/families had variability in who they want to have ACP conversations with but highlighted that it should be a trusted provider. Implementation of the guide in clinical setting not described and documentation mentioned only briefly. Conversation worksheets were provided for both the parent and adolescent/young adult.
Dombrecht, L., et al., 2020, USA [25]	Aimed to identify barriers to and facilitators of the end of life decision making process as perceived by neonatologist and nurses	Retrospective chart review	47 charts were reviewed of patients with progressive or conferred a level of fragility that would decrease lifespan	A variety of barriers and facilitators for the end of life decision making process were identified, grouped into three specific levels - case specific (uncertainty of diagnosis, specific characteristics of child, parents and HCP), decision making processes (MDT, ACP), overarching structure (i.e. privacy, policies, complex legislation)

Author, Publication Year, Study origin	Purpose	Study Design	Sample	Findings
Edwards, et al., 2012, USA [26]	To discuss how best to conduct ACP in this population through a retrospective analysis of end of life discussions involving deceased ventilator assisted patients	Retrospective chart review	47 charts were reviewed of patients with progressive or conferred a level of fragility that would decrease lifespan	About three quarters of the patients had ACP discussions prior to death, resulting in directives to forgo or limit interventions in about 50% of cases. Includes details on timing between conversations and other markers including death. Conversations often took place during acute illness i.e. in the ICU setting. The review demonstrated that unscheduled hospitalizations, ICU stays and cardiopulmonary arrests were relatively common among the cohort of children on HMV, also not perceived to be caused by their underlying condition. ACP is especially relevant for this populations
Ekberg, S., et al., 2020, Australia [27]	To develop a prompt list for pediatric palliative care	Delphi methodology	29 HCPs, 6 family participants (5 mothers, 1 mother/father dyad)	A prompt list was ultimately refined to 28 items divided into two sections (at time of referral to palliative vs end-of-life specific items), with change to conceptualize as a discussion prompt list rather than a question prompt list.
El Sayed, M., et al., 2012, Canada [28]	To explore challenges for trainees when EOL decisions are undertaken and to encourage them to reflect on how they might influence such decision making	Qualitative interviews	12 senior neonatology trainees	Study revealed six themes that reflected the challenges in EOL. These were withdrawal of life sustaining treatment based on poor outcomes, explaining no resuscitation options to parents, clarifying do not resuscitate orders, empowering families with knowledge and shared decision making, dealing with different cultures and managing person internal conflict. Most difficulty occurred in early stages of training and over time reported good knowledge of EOL care process.

				Found security and confidence from working in multidisciplinary teams.

Author, Publication Year, Study origin	Purpose	Study Design	Sample	Findings
Ewing, K., 2020, USA [29]	Educational article reviews pediatric palliative care, advance care planning and goes over components of three commonly used pediatric focused advanced directives	Educational article	n/a	Describes in depth pediatric focused advance directives: Five Wishes, My Wishes, Voicing my CHOICES. The article outlines age related limitations in child involvement in decision making along with barriers to advance care planning for nurses such as lack of formal palliative care involvement, emotional and psychological confusion or resistance and lack of education.
Fahner, J., et al., 2020, Netherlands [30]	To identify how parents envision the future when caring for their seriously ill child	Interpretative qualitative study	20 parents of 17 seriously ill children	Four main themes were identified when parents were asked to envision the future of their child: there is a focus on the near future, future perspectives are intertwined with experiences in the present and past, future perspectives range from a disease related orientation to a value based orientation and there is "no sharing without caring". Parents reported needing acknowledgement of their challenging situation, care task and their expertise in caring for their child as a precondition to feel comfortable sharing their thoughts about the future of their child.
Fahner, J., et al., 2020, Netherlands [31]	This study described the development, and pilot evaluation of the Implementing Pediatric Advance Care Planning Toolkit (IMPACT)	Qualitative pilot study	27 children with LLI, 41 parents of children with a LLI, 18 HCPs who work with children with LLI (11	Through a detailed engagement process, the designed tool IMPACT was able to provide a holistic + caring approach to ACP while giving children a voice and caring for their parents; the tool involved development of comprehensive training guidelines and conversation guides. Did not discuss documentation and intervention was aimed at individuals, system factors were not addressed.

			physicians and 7 RNs)	

Author, Publication Year, Study origin	Purpose	Study Design	Sample	Findings
Fraser, J. et al., 2009, UK [32]	Describes the UK context to pediatric ACP and discusses end of life planning in children with life limiting conditions	Educational article	n/a	Highlights some of the barriers and historical issues with pediatric palliative care and end of life planning – difficulty predicting prognosis, difficulty of “raising the issue”. Describes and provides the "Wishes Document" which was developed previously (2007) for as pediatric ACP guide/documentation and discusses the components of the document as well as how best to use it
Gallagher, A., et al., 2018, UK [33]	Introduces the ADVANCE toolkit featuring a values-based framework that aims to help caregivers who work with young people who have learning disabilities, including nurses, social workers and care assistants, develop their confidence and skills in end of life care planning	Educational article	n/a	Provides context of end of life care plans for young people who have learning disabilities. Highlights the unique considerations for advance care planning in this population and discusses the ADVANCE framework that provides recommendations for how to have discussions about planning end of life care with young people with learning disabilities and their families. The ADVANCE framework highlights key points, for example, not to make assumptions about the abilities and wishes of care for recipients and families, respect the worth of each individual in the care context, be sensitive to people’s vulnerability towards end of life and strive to maximize information giving and understanding about end of life care options.
Harmoney, K., et al., 2019, USA [34]	To determine the relationship between PPC involvement, ACP and circumstances of death for pediatric patients.	Retrospective chart review	Pediatric patients who died between a period of time at a tertiary hospital	About half of pediatric deaths had PPC involvement prior to death, those with PPC involvement were more likely to have had ACP address before death and GOC documented, code status form completed, DNR code status and hospice involvement at the time of death. Patients with chronic complex conditions were more likely to receive PPC.

Author, Publication Year, Study origin	Purpose	Study Design	Sample	Findings
Harrop, et al., 2018, UK [35]	To explore multidisciplinary clinician perceptions about perceived barriers and strategies to improve ACP provision	Educational article	n/a	Discusses the reasons why ACPs are important (location of death), when they should be performed (a time that best suits the family), how (give warning), and by who (with expertise and relationship with family). Also discusses practical pitfalls to consider including how instructions work in various settings, shared understanding of the decision, distribution, parents changing mind, required reconsideration of ACP. Article involved perspective of parents and HCPs. The article does not discuss documentation
Noyes, et al., 2013, UK [36]	To develop and evaluate the My Choices booklets for use by parents and children to facilitate thinking and engaging with future care planning	Explanatory model	13 HCPs, 12 parents (3 bereaved; 11 mothers + 4 fathers, 11 children)	Parents and children had varied preferences for interacting with the booklets, some used them to produce detailed written ACP plans, others preferred to use the booklet to spark thinking and others found it too difficult to think about the concepts in the booklet. Few families had previously shared their wishes with professionals. Professionals had varied confidence in ability to conduct ACP discussions. The My Choices booklet was revised based on feedback received. Other topics mentioned included transition to adult services, use of the ACP after the child dies, importance of considering preferred location of care, sharing of information between involved parties.

Author, Publication Year, Study origin	Purpose	Study Design	Sample	Findings
Heckford, et al., 2014, UK [37]	Was to review advance care planning for children with life threatening or life limiting conditions in our local area	Retrospective case note review	42 sets of case notes were reviewed in relation to 20 children	In one-quarter of cases there was no documented discussion of approach to end of life care, in 25% of cases there was no evidence of an ACP, half of families were not offered a choice regarding location of care and location of death despite a vast majority of those who were offered a choice choosing not the hospital, all those who were not offered a choice died in hospital. Wide variability of what components were documented when an ACP had been completed. No evidence of specific EOL discussions with the children themselves.
Hein et al., 2020, Germany [38]	Identifying key components of pediatric advance care planning through direct discussions with all involved parties	Qualitative design with a participatory approach	9 bereaved parents (6 mothers and 3 fathers) of children of different ages (2-16 years) with LLI. 14 HCPs	Key elements for ACP mentioned included discussions (engaging with a person of trust), documentation (recommended brief form for emergencies and larger advance directives for all situations), implementation (barriers including emotional barriers, disagreements between parents and professionals, difficulties with emergency services), timing of discussions (consider parental readiness, repeated at regular intervals), consideration for emerging needs and increasing awareness of families over time, involvement of children and adolescents when possible recognizing this can be challenging. Clinicians were more concerned with documentation while families were concerned with process.

Author, Publication Year, Study origin	Purpose	Study Design	Sample	Findings
Hughes, 2019, UK [40]	To understand the views and experiences of young people(s), their parents/caregivers and their healthcare providers.	qualitative	9 bereaved parents (6 males, 3 females) who received PPC, 14 HCPs	The qualitative study identified 6 themes related to ACP: understanding of ACP, practical implementation of ACP, communication/education/training for HCPs, relationships, organizational structure and culture. Experiences of young people engaging in ACP were mixed but generally positive. Facilitators included initiation of ACP when patients are in their mid-teens and conditions are stable, triggers to start an ACP discussion and standardized documentation. Barriers include poor communication, relationships with significant power differential, organizational structures and cultures which are inflexible, lack of funding, and lack of affordable training for HCPs
Jack et al., 2018, UK [39]	To explore health care professionals' views and experiences of ACP and the documentation within a pediatric setting	Qualitative, naturalistic interpretative design	21 HCPs	Showed value of process and documentation. Two major themes were identified: timing of planning conversations (waiting for relationship to build with family, introduction of parallel planning, avoiding a crisis situations) and supporting effective ACP conversations (where to have the conversation, introducing the conversation, how to approach the topic including both ACP and documentation)
Jordan et al., 2018, UK [41]	Provides a review of the unique components of pediatric palliative care.	Educational article	n/a	Lists 10 tips for palliative care providers to know when to provide palliative care to children, discussion of ACP is limited to talking about how parents are typically given discretion to make decisions for their child, the importance of involving children in a developmentally appropriate manner, and the importance of understanding the families' narrative and their definition of QOL for guiding goals of care discussions

Author, Publication Year, Study origin	Purpose	Study Design	Sample	Findings
Katz et al., 2020, Australia [42]	To assess clinicians' experience, attitudes and confidence with advance care planning at a quaternary pediatric referral center using a learning needs survey and then apply this information to develop and examine the feasibility of simulation based education for this topic.	Mixed methods	157 surveys from clinicians who work with LLI across a quaternarypediatric referral center	Only 40-45% of respondents felt comfortable to know when to hold an ACP discussion and what to include in the conversation. ACP discussions occur most frequently when death is imminent rather than in advance. A variety of barriers were identified, including differences between patient/parent and clinician understanding of prognosis, lack of parental readiness to have conversation, clinicians unsure of when is the right time, clinician uncertainty about prognosis, unrealistic parent expectations and clinician concerns about taking away hope. Participants who took part in the educational ACP simulation reported a high satisfaction rate with 90% feeling confident to hold ACP discussions after participating
Knochel et al., 2021, Germany [43]	Aimed at developing a pediatric ACP program meeting specific needs of children, parents and professionals	Qualitative participatory constellation analysis	9 bereaved families (6 mothers, 3 fathers) of LLC, 18 HCPs	Important areas of focus for pediatric ACP included the children's quality of life and individualized, interdisciplinary collaboration throughout the illness trajectory. The program was conceptualized in a modular design with fixed starting modules (to build a trusting relationship and frame the process) and at the end (to summarize results and prepare implementation), with the rest of the main discussion topics being flexible (about the child, emergencies, disease specific, EOL care). General themes that were important to consider included timing, communication, engaging children, structural issues, documentation.

Author, Publication Year, Study origin	Purpose	Study Design	Sample	Findings
Liberman et al., 2014, USA [44]	To explore parents' and caregivers' experience, knowledge and preferences regarding advance directives for children who have chronic illness.	Qualitative, Prospective cross sectional survey	307 parents from a hospital primary care pediatric clinic, pulmonary clinic and ED	Convenience sample at one hospital site. Previous advance directive experience was low amongst the cohort. Previous AD knowledge was significantly more likely among parents and caregivers with high educational degrees, parents/caregivers that were English speaking. Interest in creating an advance directive was ~50% of the study population and more likely among families who had more frequent ED visits over the past year.
Loeffen et al., 2017, Netherlands [45]	Aimed to develop and test a functional individualized pediatric palliative care plan that covers physical, psychological, spiritual and social functioning.	Retrospective and prospective pilot	Working group of 28 individuals with "strong multidisciplinary character"	The final version of the created individualized pediatric palliative care plan included 5 domains (IPPCP data, basics, social, psychosocial + spiritual, physical care). Comprehensive documents were found during the pilot phase. Comprehensive advance care plan and documentation component not explored fully. Limited involvement of parent perspectives relative to the number of healthcare providers involved.
Lord et al., 2020, Canada [46]	To explore the experiences of bereaved family caregivers with ACP for CMC	Qualitative	13 bereaved family caregivers of CMC whose deaths had occurred in the past five years	Purposeful sampling. Themes identified from parent interviews included structures of care, ACP process - including the patient and family context (existing medical and technology needs, prognostic uncertainty, goals for child, perception of quality of life, parent as expert, past experiences with life-threatening events) and ACP discussions (family-centered pace and timing, comfortable setting, appropriate people included, compassionate approach), and end-of-life care outcomes (relative shock, location, multiple losses, grief and bereavement).

Author, Publication Year, Study origin	Purpose	Study Design	Sample	Findings
Lotz et al., 2012, Germany [47]	To systematically review the empirical literature on pACP for severely ill children and adolescents to outline the current practice and effects of, as well as perspectives on pACP	Systematic review	n/a	Review identified only 3 pACP programs, two of which were informed by adult programs. Major features of pACP programs include discussions between families and HCPs, advance directives, involvement of chaplain and other providers as required. Program evaluation variable, only 1 program was studied using an RCT. Early data suggests that pACP can successfully be implemented and is perceived as helpful. Significant challenges include negative reactions from emergency services, schools and the community.
Lotz et al., 2015, Germany [7]	Aimed to investigate the attitudes and needs of health care professionals with regard to pediatric advance care planning	Qualitative interview study	17 interviews with healthcare professionals caring for severely ill children/ adolescents (9 physicians, 6 nurses, 2 social professionals)	Perceived challenges with ACP included professionals' discomfort, uncertainty regarding EOL and ACP, conflicts between physicians and non-medical care providers, avoidance of responsibility for treatment limitations according to the advanced directed. Perceived benefits included providing an action plan for everyone, ensuring that patient/parent wishes are respected. Important requirements included repeated discussions and shared decision making with family, qualified facilitator who ensures continuity through process, multi-professional conferences, continued education.

Author, Publication Year, Study origin	Purpose	Study Design	Sample	Findings
Lotz et al., 2017, Germany [9]	Aimed to investigate parents' views and needs regarding pediatric advance care planning	Qualitative	11 bereaved parents of 9 children	Purposeful sampling. Parents reported mixed feelings about ACP, finding it helpful but that it is difficult to engage in. They prefer a sensitive, individualized and gradual approach with issues of hope and quality of life being most important. There are a number of non-medical concerns parents have that they want to discuss as part of ACP. Written advanced directives were felt to be less important but parents acknowledged that medical emergency plans are viewed as necessary in particular cases. Continuity of care and information was also important, as was a continuous contact person to facilitate ACP.
Lyon et al., 2009, USA [48]	To test the effectiveness of a model of family/adolescent centered advance care planning for adolescents living with HIV and their families for increasing congruence and quality of communication while decreasing decisional conflict	Randomized controlled trial	38 dyads (aged 14 to 21 year) with HIV/AIDS and surrogates/families aged >21 years	Those participating in the ACP intervention (compared to the health lifestyle control group) reported feeling significantly better informed about end-of-life decisions, that their attitudes and wishes were known by the interviewer, and that the quality of the discussion was good to excellent. There was higher congruence between adolescent and adult surrogate in the intervention group vs control group when it came to advanced directives in certain scenarios.
Lyon et al., 2009, USA [49]	To develop, adapt and ensure feasibility, acceptability and safety of the Family/Adolescent Centered (FACE) Advance Care Planning intervention	Randomized controlled trial	38 dyads (aged 14 to 21 year) with HIV/AIDS and surrogates/families aged >21 years	Participating adolescents and their guardians/surrogates tended to rate sessions positively. Retention rate was high (93%). Results were compared to a health-living control group

Author, Publication Year, Study origin	Purpose	Study Design	Sample	Findings
Lyon et al., 2009, USA [50]	To determine the safety of engaging HIV positive adolescents in a Family Centered Advance Care (FACE) planning intervention	Randomized controlled trial	38 dyads (aged 14 to 21 year) with HIV/AIDS and surrogates/families aged >21 years	Participants in the ACP intervention completed advanced directs significantly more than controls (95% completion rate). No increase in anxiety or depression scores following the intervention, and those participating maintained quality of life. However, families perceived their adolescents as worsening in school and emotional quality of life at 3 months following the intervention compared to controls.
Lyon et al., 2014, USA [51]	To test the feasibility, acceptability and safety of a pediatric advance care planning intervention, Family Centered Advance Care planning for Teens with Cancer (FACE-TC)	Randomized controlled trial	30 Adolescent (age 14-20 years) / parent dyads with a cancer diagnosis	High enrollment rates, perfect attendance rates, 93% retention at 3 months mark suggested high acceptability of the intervention. The majority of adolescents rated the intervention as worthwhile (which increased over time) and all adult surrogates felt it was worthwhile (100%). Adolescent anxiety decreased significantly following the intervention; high QOL and low depression scores were maintained. Advanced directives were easily accessible in medical records following the intervention.
Martin et al., 2019, UK [52]	To compare how planning has developed over the 5 years across a range of children's health care services in a single UK city	Retrospective chart review	n/a	The proportion of records containing evidence of a prognostic discussion rose from 73% to 91% over the 5 year period studied. The Child and Family Wishes tool was found to be used regularly in the second review period, and ACP plans were more detailed in the later review period. The proportion of cases where preferred location of death matched the actual location of death was stable at ~50%

Author, Publication Year, Study origin	Purpose	Study Design	Sample	Findings
Mitchell et al., 2013, UK [53]	Aimed to identify and compare pediatric ACP documents that are in use within UK hospitals with a PICU	Mixed methods survey	24 of the 28 UK PICUs participated,	Survey suggests there is currently wide variation in the availability and nature of formal pediatric advance care planning documents in the UK. Just over half (58%) of responding PICUs had formal ACP documents that were currently in use, only half of which provided details about the document in use. There was wide variability in the presentation, content and intended use of ACP documents. Document characteristics included location where ACP to be kept, review dates, seizure management, infection management, other acute deterioration plan, resuscitation plan, preferred place of care, family wishes, end of life-wishes, organ donation, clinician contacts, patient/parent leaflet, ambulance crew instructions.
Myers et al., 2018, Canada [54]	To provide evidence regarding tools and/or practices available for use by healthcare providers to effectively facilitate advance care planning conversations and/or goals of care discussions	Systematic review	n/a	Several included studies reported positive outcomes however there is a lack of consistent patient outcome evidence to support any one clinical tool. Only 3 pediatric studies identified. Documentation tools included FACE, Footprints and Respecting choices. Important identified components of ACP discussions included provider education and communication skill development, standardized and accessible documentation, quality improvement initiatives, system wide coordination. Limited pediatric tools. Limited pediatric studies. Variable legal contexts to consider based on jurisdiction. Variability in tools and outcomes makes data assessment challenging.

Author, Publication Year, Study origin	Purpose	Study Design	Sample	Findings
Orkin et al., 2020, Canada [55]	To develop in depth understanding of the ACP experiences from the perspectives of both parents and health care providers of CMC	Qualitative	14 mothers of a CMC and 11 HCPs (8 physicians, 2 nurses and 1 social worker)	Major themes identified included holistic mind set, discussion content (including beliefs and values, hopes, goals, quality of life), communication enhancers (partnerships in shared decision making, supportive setting, early and ongoing conversations, consistent language and practice, family readiness, provider expertise and comfort in ACP discussions) and the definition of ACP. Many caregivers noted they had never heard the term ACP.
Pao et al., 2018, USA [56]	Explores considerations for talking with adolescents about death and dying	Review article	n/a	Review of components related to ACP with adolescents, including developmental considerations, when, how, who, why to conduct ACP.
Rishel, C., 2010, USA [57]	To describe the process of parental decision making for do not resuscitate or to withdraw life support in pediatric bone marrow transplant	Thesis	7 parents who were involved in end of life decision making for their child	The process that parents used to navigate having to make end of life decisions for their children included four major themes: developing trust, commitment to seeing it through, facing their worst fear and acceptance of self.
Sidgwick et al., 2019, UK [8]	Examines the complexities of decision making in children with LLCs who are admitted to pediatric critical care areas	Review article	n/a	Discusses the importance of parallel planning for children with life-limiting conditions admitted to pediatric critical care units - the concept of planning for the worst but hoping for the best, and outlines some of the challenges that parallel planning presents for clinicians and families. Recommended solutions include early identification of patients who may benefit in parallel planning discussions and creation of ACPs, education regarding parallel planning in medical education curricula, consideration for ACP routinely across pediatric healthcare settings.

Author, Publication Year, Study origin	Purpose	Study Design	Sample	Findings
Smith, S., 2017, USA [58]	To evaluate the use of an advance directive document as a guide to initiate communication about ACP with standardized young adult patient in a simulated clinical setting	Quantitative survey	18 nurse providers from a large metropolitan teaching hospital who care for children, adolescents and young adults with life limiting conditions	The nurse providers who participated in the simulation session reported significantly increased self-confidence in ACP following the session (including confidence in ability to initiate and discuss ACP with you patients and acknowledgement that they have the skills needed to have an ACP discussion with their patients). Participants rated the simulation experience as positive and very few had previously had any training in pediatric ACP. It was also noted participants recognized how helpful it was to use the Voicing my Choices document as a guide for communication.
Snaman et al., 2019, USA [59]	Described the development of a novel tool that quantifies the relative importance of numerous factors considered by AYA patients with cancer, their parents and health care providers when choosing between treatment options	Discrete choice experiment	Five AYA and their parents with two oncology healthcare providers	The study identified nine attributes for assessment of preference. It then describes development of the MyPref, a decision making and communication tool for AYA with relapsed/progressive cancer. The MyPref provides an opportunity for AYA to identify their preferences related to the nine attributes. The study is ongoing with an anticipated sample population of 50 participants.

Author, Publication Year, Study origin	Purpose	Study Design	Sample	Findings
Stark et al., 2008, Australia [60]	To understand the circumstance of inpatient deaths at a tertiary pediatric hospital and current practices regarding the timing and documentation of discussions concerning the withholding and withdrawing of life sustaining medical treatment	Retrospective chart review		A large majority (84%) of inpatient deaths occurred in an intensive care setting, and 74% of patients had underlying life-limiting/threatening condition. There was documented family involvement in the decision making process in 98% of the cases. 83% of discussions about EOL care took place on the day of death itself or in the week prior to the child's death. The content, clarity and accessibility of documentation of the conversations varied greatly. Study methods mean discussions that were not documented in the medical record was missed, high acuity neonatal population compared to other hospitals, records from referring hospitals were not available for review
Tatterton, M., 2018, UK [61]	To review the role of advance care planning in managing distressing symptoms in life limiting conditions	Review article	n/a	Overview of ACP practices and anticipatory symptom management. Reviewed common distressing symptoms for children with life limiting conditions and relevant definitions. Recommendations to include anticipatory symptom management and prescription with ACP.
Thompkins et al., 2021, USA [62]	To examine the effect of family centered pediatric advance care planning intervention for teens with cancer (FACE-TC) advance care planning on families' appraisals of their caregiving, distress and strain	Randomized controlled trial	126 AYA and family dyads	Participants in the intervention group reported significantly increased positive caregiving appraisals following the intervention compared to the control group. No differences found between groups with respect to caregiver strain or distress. Families benefited overall from participation in the intervention. Lower participation than hoped for, no data on the adolescent participants in this report nor the impacts of the intervention on the creation of the ACP documentation

Author, Publication Year, Study origin	Purpose	Study Design	Sample	Findings
Toce et al., 2003, USA [63]	Described the model of pediatric palliative care that focuses on advance care planning and care coordination through the FOOTPRINTS model	Descriptive article	Pilot program was 11 neonates	Describes the FOOTPRINTS model for advanced care planning and care coordination for children and their families with life limiting conditions. Program had three components: clinical program for advance care planning and coordination; research and evaluation; and education. The article outlines the evaluation, recommendations and application.
Valluzzi et al. [64]	Illustrative care scenarios, a sample plan and policy recommendations are provided	Narrative article	n/a	Narrative article which provides fictional case examples and a sample emergency response plan to be used by educational/allied health providers in the community for a child with special healthcare needs who reasonably could experience a life-threatening health emergency in the community setting.
Van Driessche et al., 2021, Belgium [65]	To describe the process to evaluate the effectiveness and implementation, context and mechanisms of impact of a novel ACP program in pediatric oncology	Randomized controlled trial	n/a	Describes the study protocol for a RCT to evaluate an intervention aimed at supporting ACP conversations for adolescents with cancer and their parents. No results available yet - not expected for a few years
Verberne et al., 2021, Amsterdam [66]	To explore how parents and healthcare professionals anticipate the future of the child and family in pediatric palliative care	Qualitative	42 parents and 35 healthcare providers of 24 children receiving palliative care	Anticipation of the future was seen to come about in three forms: goal directed conversations (initiated by either parents or HCPs to ensure others could align with their perspective regarding the future), anticipated care (HCPs or parents organizing practical care arrangements for future care scenarios with or without informing the parents/HCP), and guidance on the job (short term anticipation, HCPS guiding parents as needed through difficult situations the child was currently experiencing)

Author, Publication Year, Study origin	Purpose	Study Design	Sample	Findings
Wiener et al., 2012, USA [67]	To assess and compare the usefulness, helpfulness and stress associated with reviewing a previously adapted advance care planning guide, My Thoughts, My Wishes, My Voice, in comparison with the widely used adult document Five Wishes by adolescents and young adults living with a serious illness	Quantitative	52 participants living with recurrent or metastatic cancer (26) or HIV disease (26), aged 16 to 28 years	Important components for AYA living with life-threatening illness to include in an ACP were the kind of medical treatment they want and do not want, how they would like to be cared for, information for their family and friends to know, and how they would like to be remembered. The feedback was incorporated into the creation of the document "Voicing My Choices.
Wolff et al., 2010, UK [68]	Discusses the need for person specific planning for the increasing numbers of disabled children with life limiting and life threatening conditions	Review article	n/a	Describes the personal resuscitation plan (PRP) developed in Nottingham. Discusses the need for and describes a process by which to create and document a family health personal resuscitation plan for children with life threatening and life limiting illnesses in the community. Concepts for choices are preferred place of death, organ donation, family wishes for autopsy and care of the body and funeral. PRP were seen as a positive approach as it outlines what should be done rather than what not to do as outlined by a Do Not Resuscitate order. Provides template of document.

Author, Publication Year, Study origin	Purpose	Study Design	Sample	Findings
Wu et al., 2021, Taiwan [69]	To synthesize current knowledge to identify major components and outcomes of interventions to enhance shared decision making by adolescents with cancer during and after treatment	Systematic review and meta-analysis	n/a	5 articles were identified. All interventions improved decision quality through interventions with adolescents and/or their adult ADM. No adverse events were reported. Interventions included structured sessions held 1-3x per week, weekly assignments, live-action videos, brochures, Five Wishes advance directives, follow-up counselling
Xafis et al., 2015, Australia [70]	To discuss the development of the resources, modification after reviewer feedback and findings from initial pilot implementation of the Caring Decisions handbook	Mixed methods	Handbook given to 12 parents, 2 parents of children with LLI were interviewed. 6 clinicians provided feedback via face to face interviews	Aspects parents considered included written resources, sources of information, technical understanding difficulties, inability to articulate questions, jargon, amount of information, delivery of information, ability to hear others' experiences, support/development of a trusting relationship with HCP, conflicting information and disagreement, uncertainty, involvement in decision making and the child's quality of life. Topics covered in the parent booklet resource that was developed included how do parents think about end of life decisions, life support treatment, comfort treatment and palliative care, stopping life support, stopping vs not starting treatment, different types of treatment, doing what is best, quality of life, uncertainty, who decides about life support, disagreement, religion and culture and life support, after a decision, taboo questions, what to tell other people. Low level of dissemination of the resource, clinicians reported being unsure when was the best time to use the resource, very small sample size of parents.

Author, Publication Year, Study origin	Purpose	Study Design	Sample	Findings
Yi-Moon et al., 2020, Korea [71]	Presents the process of designing workbooks for advance care planning appropriate for Korean cultural setting and describes case studies	Single inductive case studies	7 healthcare experts in pediatric palliative care (4 physicians, 2 nurses, 1 social worker); 2 patients (1 adolescent and 1 child)	Two workbooks were developed, one for children and one for adolescents. Important themes in the development process included considerations for the Korean cultural context, the role of the counsellor as a supporter being a necessary component for success of the workbook, the workbook's need to be accessible regardless of the severity of the illness, and the patient ability to choose which version of the workbook they would like to use. Limited sample size, limited ability of study to assess impact of workbook on patients and family members, family members not involved in intervention.
Zaal-Schuller et al., 2018, Netherlands [72]	To determine which elements contribute to quality of life according to parents and physicians, how quality of life is incorporated into end of life decision making and how parents and physicians discuss quality of life considerations	Qualitative	Parents of 14 children participated (3 fathers, 14 mothers), 11 physicians	Elements that contribute to the QOL of children in the study include physical well-being (absence of physical problems, comfort, basic awareness), psychological well-being (lack of awareness of impairments and limitations, ability to enjoy, feelings of fear, cognitive development), social well-being (ability to have contact with others, presence of a caring environment, ability to engage in activities without help from others, being part of a family). The way in which QOL was included in EOL decisions varied depending on the types of decisions being made and did not always fully explore QOL consideration when making end of life decisions.
Zadeh et al., 2015 [73]	This article provides guidelines on how to introduce and utilize the advance care planning guide Voicing My Choices for Adolescents and Young Adults.	Review article	n/a	Recommended to present each section in the book as a separate module and tailored to the needs of the individual AYA and their family. The article provides sample statements for specific document sections.
